# The Adjunctive Role of Nutritional Therapy in the Management of Phlegmon in Two Children with Crohn’s Disease

**DOI:** 10.3389/fped.2017.00199

**Published:** 2017-09-20

**Authors:** Andrew S. Day, Stephanie C. Brown

**Affiliations:** ^1^Department of Paediatrics, University of Otago, Christchurch, New Zealand

**Keywords:** children, Crohn disease, exclusive enteral nutrition, nutritional treatment, phelgmon

## Abstract

Crohn’s disease may be complicated by the development of penetrating (fistulizing) or structuring complications. The presentation of an intra-abdominal phlegmon or abscess with or without an associated fistula has traditionally required surgical intervention. This series of two cases illustrates a beneficial role of non-surgical management, with parenteral and then enteral nutrition playing central roles. This report further elaborates the potential adjunctive role of enteral nutrition in the management of this complication of CD.

## Introduction

Crohn’s disease (CD), one type of inflammatory bowel disease (IBD), may present at any age, with up to a quarter of cases diagnosed in childhood or adolescence (1). CD may be complicated by the development of fistulae or penetrating disease, which may lead to an extra-intestinal collection, or phlegmon. Generally, fistulizing disease is uncommon at diagnosis in children, with most children having uncomplicated inflammatory disease at that time (2). Typically, the management of a phlegmon might include drainage and/or early resection.

Exclusive enteral nutrition (EEN) is a well-established therapy to induce remission in pediatric-onset CD (3). This intervention comprises a period of a liquid diet, using a polymeric or elemental formula, with the exclusion of normal foods during this time. EEN induces remission in more than 85% of individuals and is associated with numerous other benefits, including high rates of mucosal healing, nutritional improvements, and enhanced bone health (3).

We have previously reported the use of EEN in an adolescent with an entero-vesical fistula (4) and in the management of children with peri-anal fistulae (5). A recent report indicates that EEN may have an adjunctive role in the management of phlegmon in adults with CD (6). This report illustrates the beneficial role played by EEN, in conjunction with other interventions, in two cases of children with phlegmon development early in the course of their CD.

## Report of Cases

### Case 1

A 12-year-old boy presented to a peripheral hospital with a 12-month history of increasingly severe abdominal pain over a 4-week period prior to his hospital admission. He was noted to have had poor growth and anorexia for approximately 12 months, with recent weight loss (estimated at 2 kg) and lethargy. He had otherwise been well in the past, and there was no family history of note.

On initial examination, he was found to be mildly febrile but not tachycardic, with a tender mass palpable in the lower central abdomen. Weight was 26.7 kg with height of 143.7 cm and body mass index (BMI) of 12.9. Initial bloods showed a microcytic anemia, with normal white cell and platelet counts. CRP was elevated (237 mg/l) and albumin was at the lower range of normal (35 g/l). Abdominal CT scan with oral contrast showed circumferential wall thickening over the distal 17 cm of the ileum, with an adjacent loculated collection of fluid and gas measuring at least 4 cm in diameter. There was mild proximal distention in the ileum, but no other small bowel lesions were seen, although sigmoid colon wall thickening was also evident. The child was then transferred to a tertiary pediatric center (Christchurch Hospital) for further and ongoing care.

Upon arrival in Christchurch, he was commenced on intravenous cefuroxime and metronidazole (based on his weight). Oral intake was ceased and he was started on parenteral nutrition (PN), with progressive increases in fat and protein delivery over the following 3 days. A peripherally located central venous catheter (PICC) was placed on the second day. Given the recent completion of the CT scan, cross-sectional imaging was not repeated: however, an ultrasound scan (USS) was undertaken on the day of arrival. This imaging confirmed the phlegmon, with suggestion of a fistula from the involved ileum to the collection.

During the rest of his stay, he underwent serial USSs. On Day 13, the collection was resolving, with improved ileal wall thickening. By day 19, the collection was felt to have reduced to a volume of 1 ml, with normal peristalsis of the persistently mildly thickened ileal wall. By day 25, the USS showed resolution of the collection, with further reduction in mural thickening. No new changes were evident.

He continued PN and antibiotics for 25 days. During this time, he progressively improved clinically and biochemically. The course was, however, complicated by the development of a drug reaction on day 20 (thought most likely secondary to cefuroxime). On day 25, he commenced enteral nutrition with Fortisip (Nutricia), which was well tolerated. He progressively increased his oral intake with this polymeric formula, until day 29 at which time he was on full EEN (8 × 200 ml fortisips daily, providing 2400 kcal/day) whereupon his PN was ceased. On day 33, he was discharged home, with ongoing EEN for 8 weeks in total. He was also commenced on oral azathioprine prior to discharge following standard protocol. Approximately 2 months post presentation, he underwent diagnostic upper gastrointestinal endoscopy and ileocolonoscopy. Endoscopically, there were scattered colonic ulcers with mild chronic inflammatory changes seen histologically.

Subsequently, he was seen on a regular basis with serial measurement of inflammatory markers. Three months after diagnosis, he was noted to be well, with no symptoms, had no positive examination findings, and had gained approximately 6 kg. CRP at this time was 2, along with normal albumin, platelets, and ESR. Sixteen months post diagnosis, he was well on maintenance azathioprine, with no symptoms, satisfactory growth, normal examination findings, and with normal inflammatory markers (CRP 3, albumin 36, platelets 326, and hemoglobin of 119). Ongoing follow-up continues.

### Case 2

This girl presented at 12 years of age with a history of several months of diarrhea and abdominal pain. She had anorexia with weight loss of 3 kg in the preceding weeks. At presentation, she weighed 49.25 kg and was 158.1 cm tall (with BMI of 19.7). Abdominal examination was unremarkable with no mass evident. Initial blood tests showed elevated CRP but normal platelet count. Abdominal USS showed ileal wall thickening with no extra-intestinal abnormalities. She proceeded to undergo an endoscopic assessment, with no specific abnormalities seen. Histologically, focal active gastritis and colonic eosinophilia was seen (unfortunately, ileal biopsies were not obtained).

Given her disease location, and presentation pattern, EEN was recommended and commenced on the day following her endoscopy. She started this therapy without concerns and was able to be discharged home 2 days later.

However, she re-presented with increased abdominal pain 3 days after discharge. She was found to have localized tenderness in the right iliac fossa, but no clear palpable mass evident. She proceeded to have an USS, which suggested a phlegmon. Repeat blood tests showed an elevated CRP (268 mg/l) and neutrophilia (15.1 × 10^9^/l). After reviewing these results, she was commenced on intravenous antibiotics (metronidazole, gentamicin and amoxicillin) and placed on full intravenous fluids (nil by mouth). Magnetic resonance enterography (MRE) with oral and intravenous contrast was booked and PICC line placement was arranged for the following day. MRE confirmed the USS impressions of a phlegmon, with marked ileal wall thickening extending proximally from the ileocecal valve for 20 cm. The small phlegmon was seen lying anterior to the most severely involved area of small bowel with no fistulous tract evident and no other abnormality evident on the imaging.

She was managed with PN for a fortnight, during which time her inflammatory markers fell (CRP 4 g/l at day 11). Repeat USS imaging after 2 weeks of PN showed marked improvement of the phlegmon but showed continued ileal wall thickening. She then slowly recommenced EEN and was able to establish full EEN intake orally by day 17 (at which point PN was ceased). She was also commenced on azathioprine in standard fashion and discharged home on day 18.

Over the following period of time, she was followed as an outpatient. She completed 8 weeks of EEN and resumed normal diet subsequently. After this, she remained well, with maintenance EN and azathioprine (dose at 2.5 mg/kg/day with satisfactory 6-thioguanine nucleotide levels). Follow-up included subsequent USS imaging, which showed no recurrence of collection, and routine review of serum inflammatory markers (with no increase in CRP).

Unfortunately, 18 months following diagnosis, she represented with abdominal pain and was found to have a palpable tender mass in the right iliac fossa. She was admitted and underwent a further USS followed by a second MRE, along with routine blood testing. Imaging showed recurrence of phlegmon without evidence of fistula or any other changes. She was again commenced on PN, with prompt improvements (clinically, biochemically, and radiologically) in the following 2 weeks. She restarted EEN at this time orally and quickly reached required daily volumes (7–8 fortisips daily, providing 2,100–2,400 kcal daily).

Given her recurrent presentation, surgical review was requested during this admission and arrangements were made for elective ileocecal resection. In the interim, she continued EEN. Resection was completed approximately 2 months later, with 17 cm of ileum resected along 8 cm of colon and primary re-anastomosis completed. Post-operatively she was managed with 3 months of oral metronidazole and continued oral azathioprine. Routine surveillance colonoscopy 6 months after her operation showed >6 aphthous ulcers at and distal to the anastomosis, with no stricture or other changes. She was subsequently commenced on adalimumab (standard dosage) in combination with azathioprine. Subsequently, over the following 5 years, she has been well, with no recurrent fistulizing disease, or other complication of CD.

## Discussion

These two cases illustrate the potential adjunctive role of nutritional interventions in the management of ileal CD complicated by the development of phlegmon. Both children had radiological features of a collection adjacent to the distal ileum, with a fistula demonstrated in one instance. Both children were managed in the same fashion with initial gut rest and intravenous nutrition, antibiotics, and subsequent EEN. Although the second child had recurrence of disease many months later, the acute management on this second occasion was similar and again successful. Case 1 has, however, had no further disease complications over more than 18 months of further observation. In addition to the previous case reports of EEN having roles in the management of fistulizing CD in other locations (4, 5), and a report of the benefits of EEN in the management of phlegmon in 33 adults (6), these two cases further illustrate that EEN (as a part of a managed package of conservative measures) may also have a role in this complication of CD.

The benefits of EEN in the induction of remission of active CD have been described over the last decades, with the first comparative data coming from O’Morain and colleagues in 1986 (7). Numerous studies have clearly shown that this intervention is safe and effective, with particular benefits in children and adolescents (3, 8). EEN has also been shown to enhance quality of life in children (9, 10). Consequently, EEN is now recommended as the first line management for active IBD in consensus statements (11, 12) and used in many pediatric centers in this manner.

In addition to the roles of inducing remission, EEN also clearly leads to high rates of mucosal healing, along with macro- and micro-nutritional benefits and enhanced bone health (3, 8). Furthermore, this therapy is associated with few side effects. Although the understanding of the mechanisms by which EEN acts are incomplete, data do demonstrate that EEN leads to substantial changes in the patterns of the intestinal microbiome and enhanced epithelial barrier function (3).

The adult and pediatric studies evaluating the roles of EEN in active CD have typically included subjects with luminal disease, with exclusion of those with fistulizing complications (8, 13). Previous short reports demonstrated that EEN had a role in the management of an entero-vesical fistula in an adolescent boy with active CD (4) and also in the management of two children with peri-anal fistulae (5).

Adult reports have also illustrated the benefits of EEN for fistulizing CD. A Chinese report showed that 30 of 48 adults with entero-cutaneous fistulae had complete healing after up to 3 months of EEN (14). In that series of patients, CRP level and BMI scores were associated with outcome. Similarly, EEN was successfully utilized in the management of inflammatory strictures in a series of Chinese adults (15). Thirty-five of 50 patients who completed 12 weeks of EEN achieved radiological remission, while 42 had clinical remission. Another recent report demonstrated that EEN was helpful in a group of Chinese adults with intra-abdominal abscess (*n* = 33) or stenosis (*n* = 10): EEN lead to resolution or improvement in the size of phlegmon (6). Furthermore, the use of EEN in a group of 51 adults with structuring or penetrating CD resulted in a reduction in the need for surgical intervention: one quarter of this group did not subsequently require operation (16).

The management of a phlegmon or intra-abdominal abscess in the context of CD has variously included peripheral drainage, antibiotics, and early surgical intervention (17–19). While there are no direct comparisons between these managements in the current report, the conservative nutritional approach outlined in these two cases suggests that this may be a valid option to consider. This report is limited as it involves just two children, who were assessed in slightly different manners and their outcomes reviewed retrospectively. Further careful prospective evaluation of this approach in children with this complication of CD is now required.

## Ethics Statement

This case report does not include any identifying features throughout. Accordingly, formal approval from the University of Otago Ethics Committee was not required. Written informed consent was obtained from Patient 2 and the parents of Patient 1 with their agreement to the publication of this case report.

## Author Contributions

AD prepared the initial draft of the manuscript and participated in the drafting of the work. SB participated in the drafting of the manuscript. Both authors approved the final version of the manuscript.

## Conflict of Interest Statement

The authors declare that the research was conducted in the absence of any commercial or financial relationships that could be construed as a potential conflict of interest. The reviewer JS and handling editor declared their shared affiliation.
